# Targeting G-quadruplex DNA with synthetic dendritic peptide: modulation of the proliferation of human cancer cells†

**DOI:** 10.1039/d0ra04780e

**Published:** 2020-07-14

**Authors:** Soumi Biswas, Satyabrata Samui, Apurba K. Das, Sanjeev Pasadi, K. Muniyappa, Jishu Naskar

**Affiliations:** Department of Biochemistry and Biophysics, University of Kalyani Nadia WB 741235 India jishunaskar@gmail.com; Department of Chemistry, Indian Institute of Technology Indore Indore 453552 India; Department of Biochemistry, Indian Institute of Science Bangalore Karnataka 560 012 India

## Abstract

Telomerase, a reverse transcriptase enzyme, is found to over express in most cancer cells. It elongates the telomere region by repeated adding of TTAGGG in the 3′-end and leads to excess cell proliferation which causes cancer. G-quadruplex (G4) formation can inhibit such telomere lengthening. So, stabilization of G4 structure as well as inhibition of telomerase activity is very promising approach in targeted cancer therapy. Herein, the aptitude of a synthetic dendritic peptide, C^*δ*2^–(YEE)–E (peptide 1), to target specifically the human telomeric G4 DNA, dAGGG(TTAGGG)_3_, has been evaluated. Both biochemical and biophysical techniques including gel mobility shift assay, isothermal titration calorimetry and fluorescence spectroscopy have been employed for the purpose. Circular dichroism study reveals that the targeting results an increase in thermal stability of G4 DNA. Interestingly, replacement of N-terminal tyrosine residue of peptide 1 by valine, C^*δ*2^–(VEE)–E, (peptide 2) consequences in loss of its G4 DNA targeting ability, although both the peptides exhibit comparable affinity toward double-stranded DNA. Of note, peptide 1 causes cessation of growth of human cancer cells (HeLa and U2OS) and induces apoptosis *in vitro*. But it has no significant inhibitory effect on the growth of normal human embryonic kidney 293 cells. Mechanistically, Telomeric Repeat Amplification Protocol (TRAP) assay indicates that peptide 1 effectively inhibits the telomerase activity in human cell extracts. Overall, this study demonstrates the usefulness of a synthetic dendritic peptide as an inhibitor of tumor cell growth by inducing apoptosis upon targeting the telomeric G4 DNA.

## Abstract 2

A synthetic dendritic peptide, targeting human telomeric G4 DNA, inhibits the telomerase and lessens the proliferation of human cancer cells.

## Graphical abstract

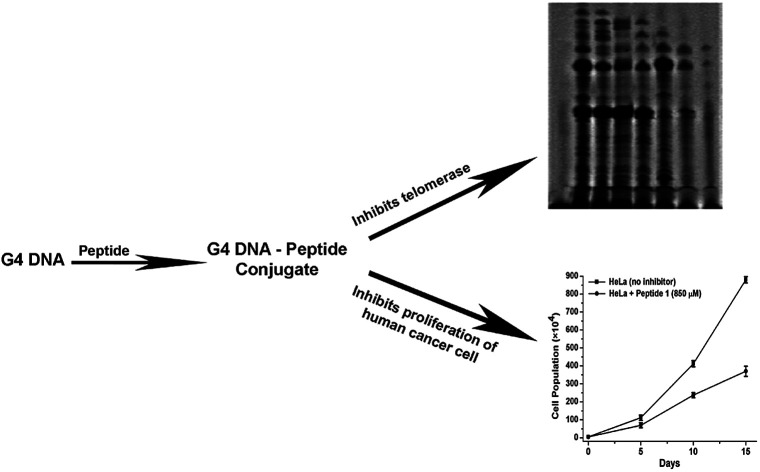

## Introduction

The G-quadruplexes (G4) are non-canonical DNA/RNA structures that play important roles in DNA replication,^1*a*^ recombination,^1*b*^ transcriptional regulation,^1*c*^ maintenance of genomic stability^1*d*^ and aging.^1*e*^ The G4 forming sequences occur throughout the human genome, but they are most prevalent in the telomeres,^2*a*^ immunoglobulin switch regions^2*b*^ and in promoters of proto-oncogenes.^2*c*^ The telomerase activity is suppressed in most human somatic cells, except in stem cells and lymphocytes,^3*a*^ but it becomes up-regulated in most tumor cells.^3*b*^ The unfolded single-stranded DNA is required for optimal telomerase activity; whereas, G-quartet formation inhibits the telomerase activity.^4^ For this reason, G4 structures are considered to be the potential biomedical targets to impede cancer progression. Consequently, an escalating interest has been grown with the development of G4 binding small molecules for therapeutic purpose.^5*a*,*b*^ In recent years, various small molecules targeting G4 structure have been designed, synthesized and evaluated for their antitumor activities against various human cancer cell lines.^6*a*–*d*^ The most interesting synthetic molecules as the telomerase inhibitor and telomeric G4 DNA binding ligand belong to the class of planar, substituted aromatic compounds. Some examples of such substituted aromatic compounds are isoquinoline alkaloids (berberine, palmatine, coralyne and sanguinarine),^7*a*^ quarfloxine,^7*b*^ RHPS4,^7*c*^ Phen-DC_3_,^7*d*^ BRACO-19,^7*e*^ tetra-substituted naphthalene diimides,^7*f*^ tri-substituted indolo[3,2-*b*]quinolines,^7*g*^ and carbazole based compounds.^7*h*–*j*^ Various metal complexes^8*a–g*^ have been found to stabilize the G4 structure. Among these, it is found that the square planar metal-complex serves as the optimal candidates for stabilization of G4 DNA. For example, Pt(ii) squares are selective and effective human telomeric G4 DNA binders and potential cancer therapeutics.^8*g*^ Several small molecules, including porphyrin,^9*a*^ carbocynamide dyes,^9*b*^ and piperine^9*c*^ are reported to bind with the quadruplex structures. Naturally occurring molecules derived from vegetables, nuts, wine, bacteria *etc.* also can serve as G4 stabilizing ligand.^10^

In this study, we explore the ability of a synthetic dendritic peptide as ligand to stabilize the G4 DNA structure. Peptides are comparatively less explored toward this end. Peptides have several advantages over proteins, antibodies and other small molecules due to their small size, ease of synthesis and purification, cell permeability, tumor penetrating ability and improved biocompatibility.^11*a*,*b*^ Previous studies have demonstrated that several dodecameric peptides stabilize the G4 structure derived from c-MYC promoter and also induce apoptosis in the cancer cells.^12*a*^ Selective recognition of human telomeric G4 DNA by designed peptides composed of glutamic acid and tryptophan residues has been reported recently.^12*b*^ In literature, there are various reports of cyclic peptides as well which have significant potential to target the G4 structure.^12*c*,*d*^ Here, we demonstrate the ability of a synthetic dendritic peptide, C^*δ*2^–(YEE)–E, to stabilize the human telomeric G4 DNA structure and also address the potential to inhibit the proliferation of human cancer cells *in vitro*.

## Results and discussions

**Scheme 1:**
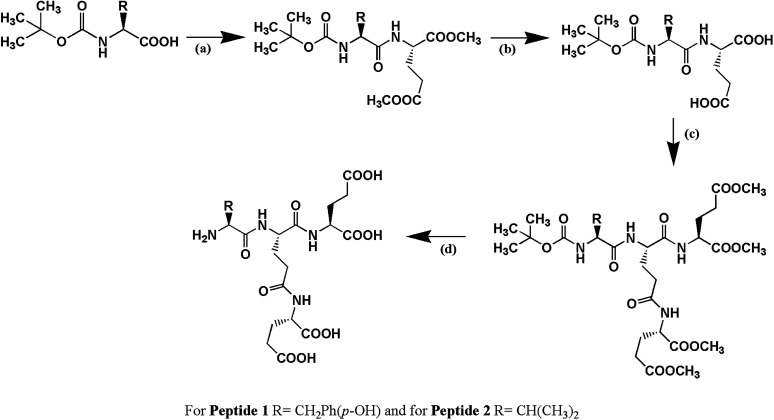
The schematic presentation of the synthesis of peptides. (a) DCC, HOBt, H-Glu(OMe)_2_, (b) MeOH, NaOH (c) DCC, HOBt, H-Glu(OMe)_2_ and (d) MeOH, NaOH followed by treatment with trifluoroacetic acid (TFA). For peptide 1, R = –CH_2_Ph(*p*-OH) and for peptide 2, R = –CH(CH_3_)_2_.

Peptide 1, C^*δ*2^–(YEE)–E, has been designed in such a way that it holds two factors (a) hydrogen bonding interaction and (b) π–π interaction simultaneously which play very important role in peptide–DNA interaction. Peptide 2, C^*δ*2^–(VEE)–E, has been designed to address whether only hydrogen bonding interaction is enough to stabilize the G4 structure. Both the peptides have been synthesized by solution phase racemization free fragment condensation strategy and final compounds have been characterized by NMR spectroscopy (^1^H and ^13^C) and mass spectrometry (Scheme 1).

### Electrophoretic gel mobility shift assay

**Fig. 1:**
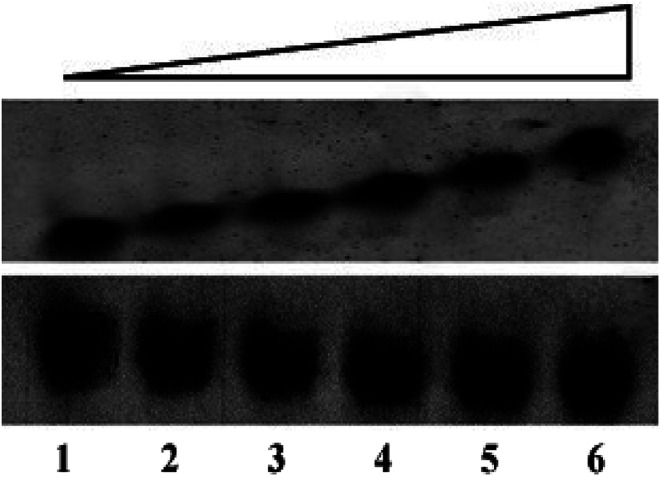
The human telomeric G4 DNA (30 μM) is incubated in the absence (lane 1) or presence (lanes 2–6) of peptides at 15, 30, 45, 60 and 120 μM respectively.The upper and lower panels represent the electrophoretic migration of G4 DNA in the presence of peptide 1 and peptide 2 respectively.

Electrophoretic mobility shift assay (EMSA) has been performed to anticipate the interaction of peptides with G4 DNA. The assay has been carried out by incubating 30 μM of G4 DNA with increasing concentrations (0, 15, 30, 45, 60 and 120 μM) of peptides for 30 min at room temperature and the DNA binding ability of the peptides is assessed by shift (retardation) in the electrophoretic migration of G4 DNA. As shown in Fig. 1 (upper panel), addition of increasing concentrations of peptide 1 led to the significant reduction in electrophoretic migration of G4 DNA (lane 2–6) compared to unbound DNA (lane 1). On the other hand, no significant reduction in the electrophoretic migration of G4 DNA is observed in the presence of peptide 2 (Fig. 1, lower panel). Thus the EMS assay indicates that the reduction in electrophoretic migration of G4 DNA in presence of peptide 1 occurs probably due to the binding interaction of peptide 1 with G4 DNA.

### Isothermal titration calorimetry study

**Fig. 2:**
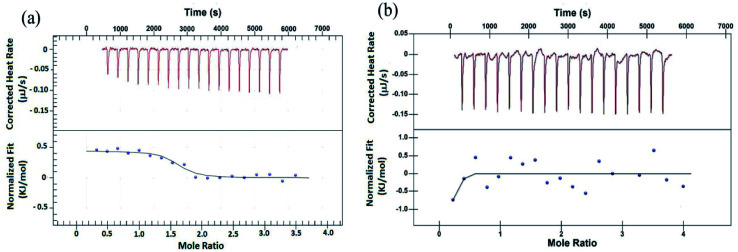
ITC profiles of titration of (a) peptide 1 with G4 DNA, (b) peptide 2 with G4 DNA at *T* = 298.15 K. The top panels of both (a) and (b) represent the amount of heat generated per sequential injection of peptides into G4 DNA solution, and the bottom panels show the integrated heat data after correction of heat dilution against the molar ratio of peptide to G4 DNA. The solid lines represent the best fitted plot.

To validate the results from EMS assay, binding of peptides to G4 DNA has been studied by isothermal titration calorimetry (ITC). ITC experiment has been carried out keeping G4 DNA (64 μM) into the cell and peptide (700 μM) into the syringe. Fig. 2 shows the characteristic ITC profiles for peptide–G4 DNA interaction. The ITC titration data of peptide 1 (Fig. 2a) yields the association constant (*K*_a_) 1.10 × 10^6^ M^−1^, enthalpy change (Δ*H*) 0.443 kJ M^−1^ and entropy change (Δ*S*) 0.1168 kJ M^−1^ K^−1^. Interestingly, under similar condition peptide 2 shows no characteristic G4 DNA binding signature (Fig. 2b). Further, to check whether these peptides bind to double stranded DNA (ds-DNA), ITC measurements have been carried out with ds26, a model ds-DNA (5′CAATCGGATCGAATTCGATCCGATTG3′). From the results, the association constants (*K*_a_) of peptide 1 and 2 with ds26 are found to be 9.60 × 10^3^ M^−1^ and 3.36 × 10^3^ M^−1^ respectively (Fig. S9†).

### Fluorescence spectroscopy

**Fig. 3:**
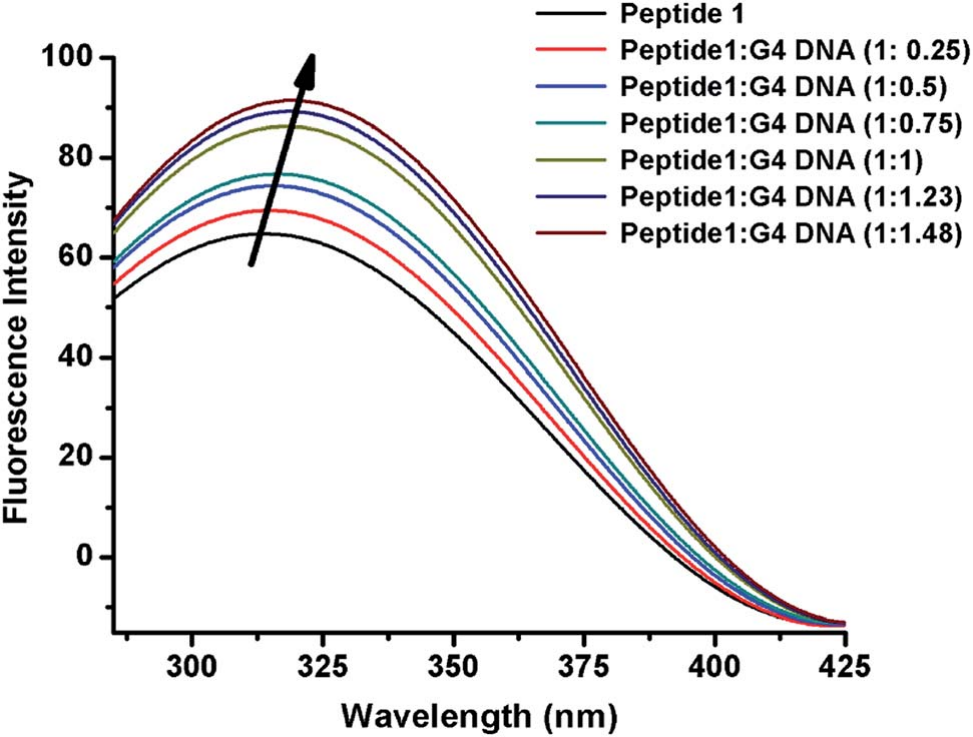
Fluorescence emission spectra of peptide 1 upon progressive addition of G4 DNA.

The interaction of peptide 1 with G4 DNA has also been monitored by fluorescence spectroscopy. Peptide 1 shows the characteristic emission band at 305 nm upon excitation at 275 nm. From Fig. 3 and S12,† it is found that the intensity of the emission band increases upon progressive addition of G4 DNA, indicating the binding interaction of peptide 1 with G4 DNA. Here it is important to note that, unlike peptide 1, peptide 2 lacks intrinsic fluorescence property, which prevents us to perform similar study with it.

### Time correlated single photon counting (TCSPC)

**Fig. 4:**
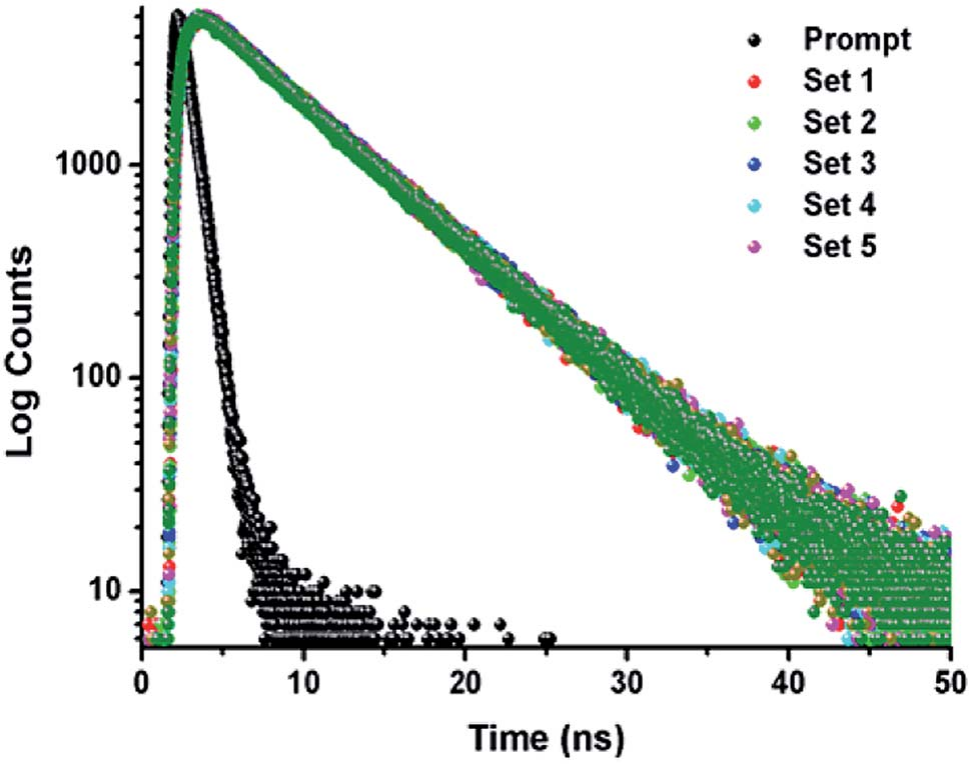
Time-resolved fluorescence spectra of peptide 1 in the absence and presence of increasing concentrations of G4 DNA in buffer (10 mM Tris and 100 mM NaCl) of pH = 7.3 at 25 °C.

The TCSPC experiment has been carried out to access the insight about the interaction behavior of peptide 1 with G4 DNA. The experiment has been done keeping the peptide 1 concentration fixed at 5.5 μM and G4 DNA concentration is varied from 0 to 10.5 μM. From the experiment, it is found that the decay pattern of peptide 1 is tri-exponential and the average life time (*τ*_avg_) is 4.93 ns. However, upon progressive addition of G4 DNA up to 10.5 μM, *τ*_avg_ value of peptide 1 decreases slowly from 4.93 to 3.09 ns (Fig. 4 and Table 1). From this result, it can be concluded that the decrease in *τ*_avg_ is attributed to the change in micro-environment of tyrosine residue of peptide 1 upon complexation with G4 DNA.^13*a*,*b*^

**Table 1:** Time-resolved fluorescence parameters of peptide 1 with increasing concentration of G4 DNA

Sample	[G4 DNA] (μM)	[Peptide] (μM)	*χ* ^2^	*τ*_1_	*τ*_2_	*τ*_3_	*q*_1_	*q*_2_	*q*_3_	Average life time *τ*_avg_ (ns)
Set-1	0	5.5	1.033	3.11 × 10^−9^	6.6 × 10^−9^	3.1 × 10^−10^	0.12	0.68	0.20	4.93
Set-2	2.5		1.004	3.08 × 10^−9^	6.61 × 10^−9^	3.0 × 10^−10^	0.12	0.67	0.21	4.86
Set-3	4.5		0.995	2.24 × 10^−9^	6.5 × 10^−9^	1.5 × 10^−10^	0.08	0.59	0.33	4.07
Set-4	8.5		1.006	1.80 × 10^−9^	6.54 × 10^−9^	8.1 × 10^−11^	0.08	0.53	0.55	3.65
Set-5	10.5		1.014	1.89 × 10^−9^	6.54 × 10^−9^	9.7 × 10^−11^	0.06	0.45	0.49	3.09

### Thiazole orange (TO) displacement assay

**Fig. 5:**
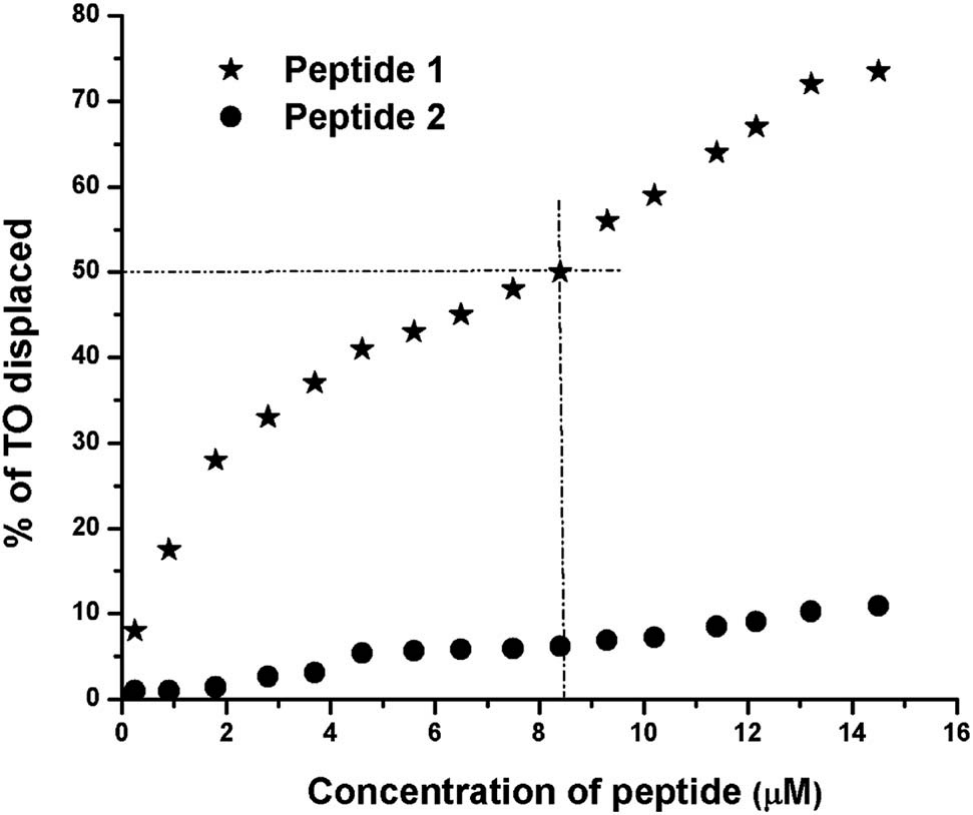
Thiazole orange (TO) displacement assay. The TO–G4 DNA complex, generated from 0.25 μM of G4 DNA and 0.50 μM of TO, is titrated with peptides 1 and 2. In each case, the peptide concentrations vary from 0 to 14.5 μM.

The TO displacement assay is extensively used to study G4-binding properties of specific ligands. Into aqueous buffer, TO exhibits week fluorescence property. But upon binding to G4 DNA, TO–G4 DNA complex exhibits a significant increase in fluorescence intensity compared to the free TO.^14^ Thus, the affinity of a given ligand toward G4 DNA can be assessed by measuring its ability to displace TO from the TO–G4 DNA complex. From the results of TO displacement assay, it is found that ∼8.4 μM peptide 1 is able to displace ∼50% TO from the TO–G4 DNA complex (Fig. 5). In contrast, an identical concentration of peptide 2 could displace only ∼6.5% TO, suggesting that peptide 1 has much higher binding affinity than peptide 2 toward G4 DNA.

### Circular dichroism (CD) spectroscopy

CD spectroscopy is a very sensitive technique to investigate ligand induced conformational changes of nucleic acids. In aqueous buffer containing Na^+^ ions, human telomeric dAGGG(TTAGGG)_3_ DNA adopts anti-parallel G4 structure which shows a strong positive band at 295 nm as well as a negative band at 260 nm followed by a shoulder at 245 nm.^15^ In presence of increasing concentrations of peptides 1 and 2 (Fig. S10†), the intensity of the positive band (at 295 nm) decreases whereas the intensity of the negative band (at 260 nm) increases. These changes indicate that the conformation of the G4 DNA gets perturbed subsequent to the binding of both peptides.

### CD melting studies

**Fig. 6:**
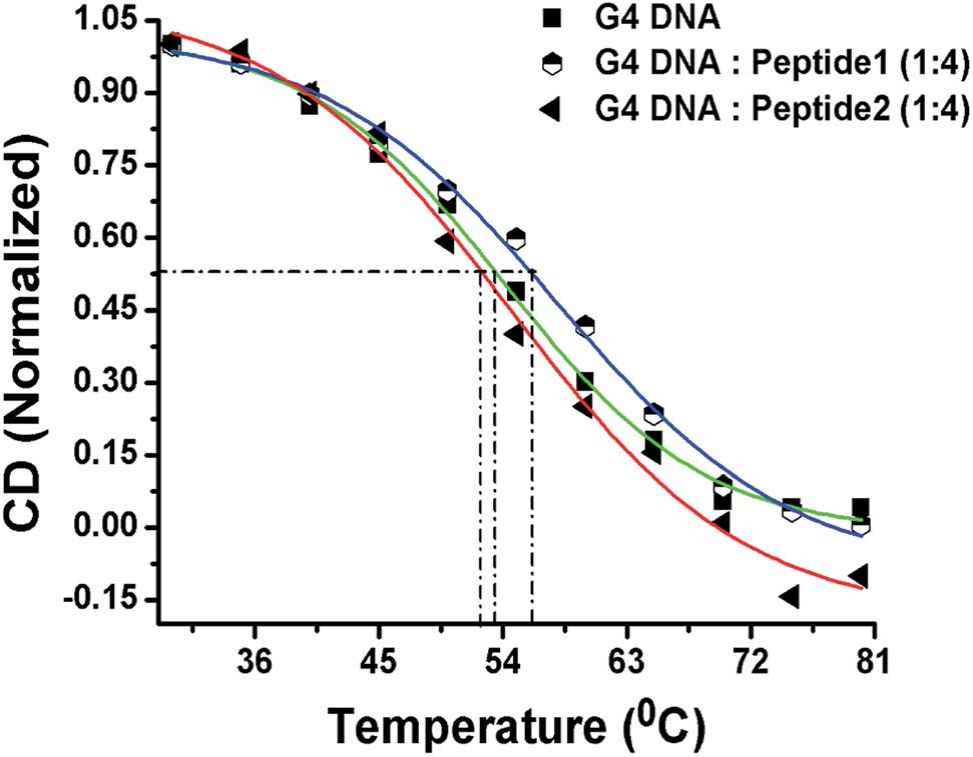
CD melting profile. Green line indicates the melting profile of G4 DNA in the absence of peptide; the blue and red lines represent the melting profile in the presence of peptide 1 and peptide 2 respectively.

Now, in order to compare the stability of human telomeric G4 DNA in the absence and presence of peptides, thermal melting study of G4 DNA is performed using CD spectroscopy. The melting curve is constructed by plotting the molar ellipticity (at 295 nm) against temperature.^16^ The melting profile of G4 DNA in the presence of peptide 1 reveals (Fig. 6) an increase in the melting temperature (*T*_m_) of ∼3 °C compared to free G4 DNA. On the other hand, the *T*_m_ value is found to decrease ∼1 °C in the presence of peptide 2. Thus, CD melting studies clearly indicate that the peptide 1 increases the thermal stability of G4 DNA but peptide 2 doesn't.

### Peptide 1 inhibits cellular telomerase

**Fig. 7:**
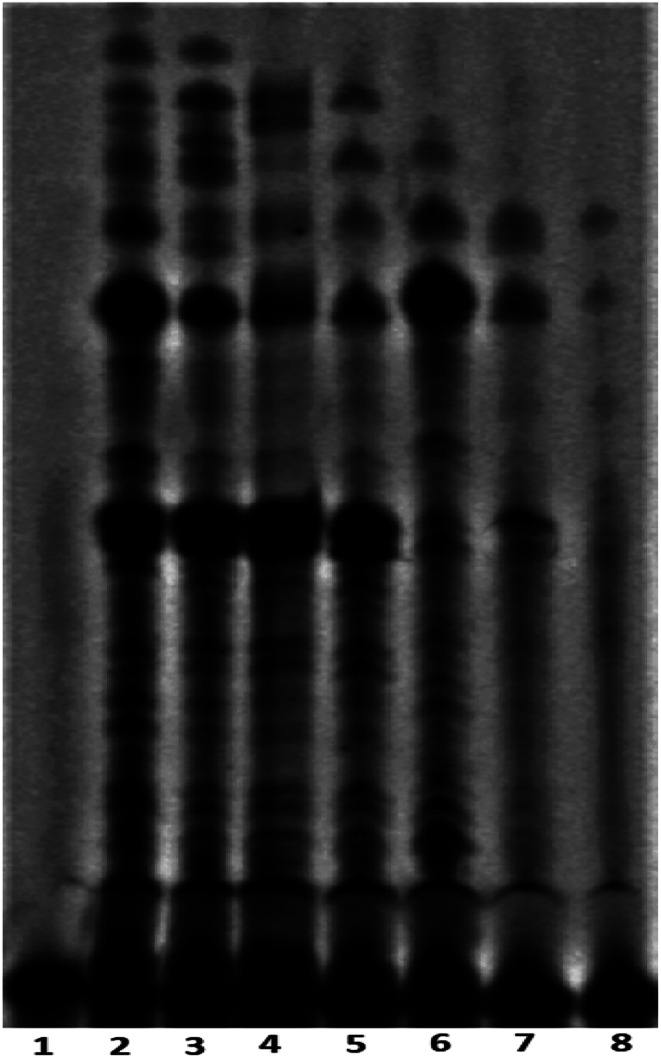
Telomerase inhibition assay with peptide 1, lane 1: negative control (in absence of telomerase enzyme and peptide 1), lane 2: positive control (in absence of peptide 1), lanes 3–8 with increasing concentrations of peptide 1 (from 0.25, 0.5, 0.75, 1.0, 1.5 and 2.0 mM respectively).

The telomerase activity is found to be up-regulated (nearly 90%) in cancer cells compared to normal differentiated somatic cells.^17^ To gain a better understanding of the mechanism of action exerted by peptide 1, the mode of telomerase inhibition has been assayed using PCR-based Telomeric Repeat Amplification Protocol (TRAP) assay with HeLa nuclear extract.^8*a*,*b*^ The TRAP assay has been performed using the concentrations of peptide 1 ranging from 0.25 to 2.0 mM. As shown in Fig. 7, the increasing concentrations of peptide 1 (lane 3–8) inhibit the telomerase activity in a dose-dependent manner *in vitro*.

### Cytotoxic effect of peptide 1 on cancer and non-cancer cells

**Fig. 8:**
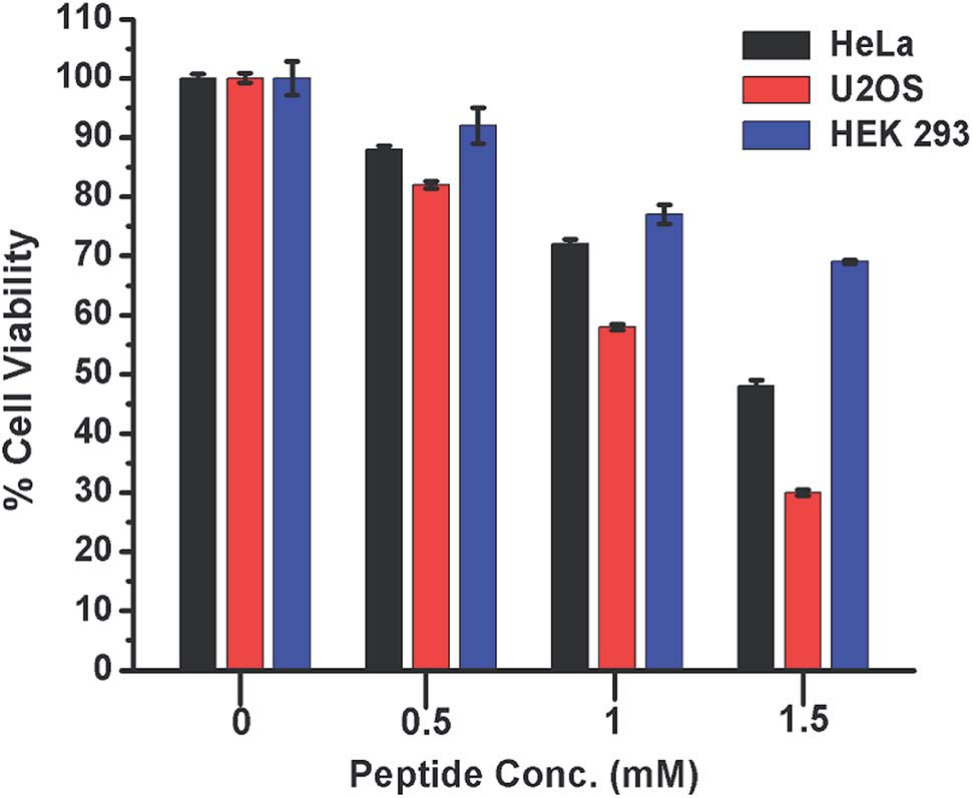
Cytotoxicity of peptide 1 on cancer and non-cancer cells. The effect of peptide 1 on the cell viability is measured by MTT assay after 72 h of exposure. In this assay the peptide concentration is varied from 0 to 1.5 mM.The values are mean of three independent experiments.

From the foregoing results, it is evident that peptide 1 confers the thermodynamic stability to G4 DNA. The ligands, those stabilize the G4 DNA display the anticancer property.^5,18^ Therefore, to check whether peptide 1 could modulate the proliferation of cancer cells, HeLa and U2OS cells have been selected for the cytotoxicity analysis *in vitro*. The human embryonic kidney (HEK 293) cell line served as a negative control. The assay has been performed by treating the cells with various concentrations of peptide 1 (0, 0.5, 1.0 and 1.5 mM) for 72 h. As the result, it is found that peptide 1 exhibits much higher cytotoxicity on cancer cells compared to non-cancer cell under the similar conditions (Fig. 8). The IC_50_ values are1.48 mM and 1.1 mM respectively for HeLa and U2OS cells. Interestingly, at 1.5 mM concentration of peptide 1, ∼70% of HEK 293 cells are found to be viable. Further, to examine the effect of peptide 1 on cancer and non-cancer cells, phase contrast microscopic study has been performed. The study reveals that the effect of peptide 1 to inhibit the proliferation of HeLa cells and subsequent cell death is much higher compared to HEK 293 cells (Fig. S11†). These results suggest that peptide 1 exerts significant cytotoxic effect on cancer cells, but has very little effect on the non-cancer cells.

### Long-term cell viability assay

**Fig. 9:**
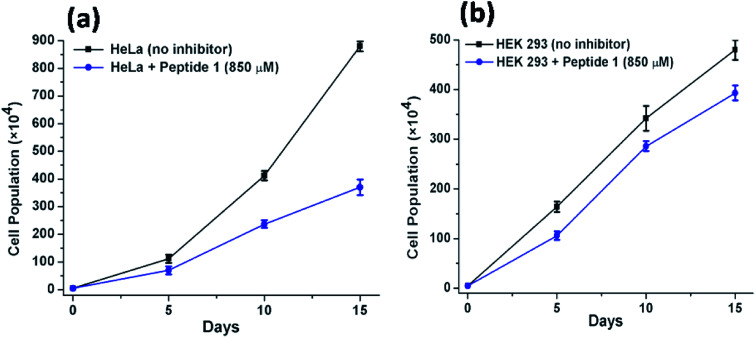
Effect of peptide 1 on the proliferation of (a) HeLa and HEK 293 (b) cells over time.

Further to investigate the cytotoxic and growth inhibitory effect of a sub-inhibitory dose of peptide 1, the cell proliferation assay has been performed over a longer period of time. For this purpose, the HeLa and HEK 293 cells have been treated with 850 μM of peptide 1 for 15 days. Upon treatment, it is found that (Fig. 9) the proliferation of HeLa cells lessens from (880 ± 18) × 10^4^ to (370 ± 28) × 10^4^. In contrast, the proliferation of HEK 293 cells under similar condition is found to lessen from (480 ± 20) × 10^4^ to (393 ± 15) × 10^4^. Thus, the studies clearly establish that the inhibition efficiency of peptide 1 on cancer cells is significantly higher than that in the normal cells regardless of dose and time of treatment.

### Study of mechanism of cell death

**Fig. 10:**
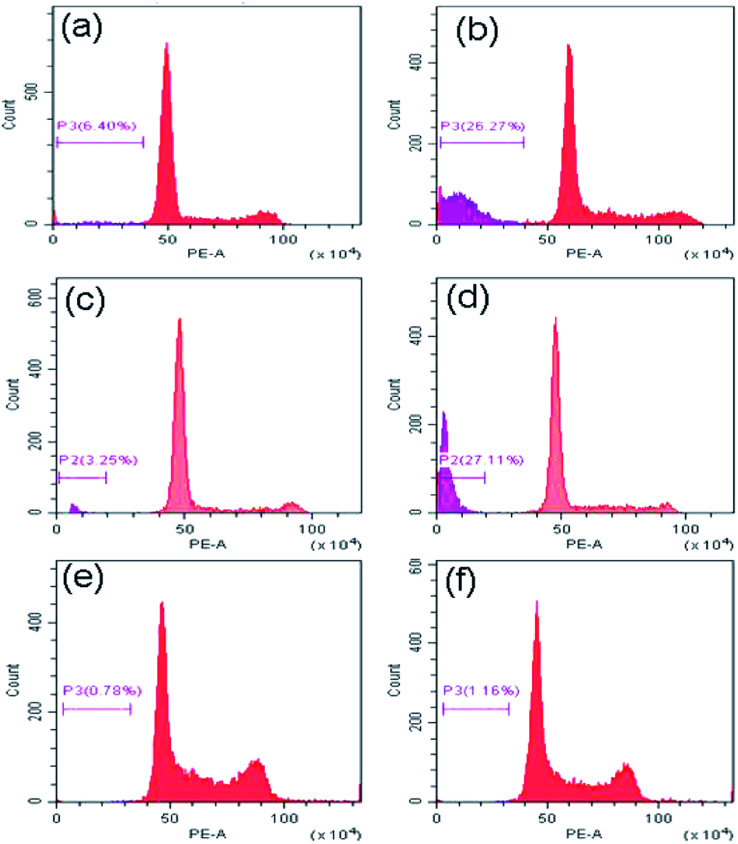
Cell cycle analysis to determine the percentage of cells in sub-G1 (apoptotic) region. (a), (c) and (e) are the HeLa, U2OS and HEK 293 cells alone, (b), (d) and (f) are the HeLa, U2OS and HEK 293 cells after treatment with peptide 1 for 48 h.

Now to ascertain, whether peptide 1 induced cell death is due to apoptosis, the cell cycle analysis has been performed by staining HeLa, U2OS and HEK 293 cells with propidium iodide. The results (Fig. 10) show that the sub-G1 population in the treated cells increases significantly in case of HeLa and U2OS with respect to untreated control which clearly indicates the cell death is through apoptosis.

## Conclusion

In this study, we describe the interaction of two synthetic dendritic peptides with human telomeric G4 DNA. The results establish that the peptide 1, C^*δ*2^–(YEE)–E, containing tyrosine as the N-terminal residue binds robustly to G4 DNA. The binding affinity is drastically reduced in peptide 2, C^*δ*2^–(VEE)–E where N-terminal tyrosine residue has been replaced by valine. CD measurements reveal that binding of peptide 1 increases the thermal stability of G4 DNA. Importantly, peptide 1 exhibits significant telomerase inhibition property along with profound cytotoxic effect selectively on cancer cells compared to non-cancer cells. These results underscore the utility of a short, synthetic peptide as potential anti-tumor agent targeting telomeric G4 DNA.

## Conflicts of interest

There are no conflicts of interest to declare.

## Supplementary Material

RA-010-D0RA04780E-s001
