# Supplementary material for: Targeting G-quadruplex DNA with synthetic dendritic peptide: modulation of the proliferation of human cancer cells
Source: RSC Adv. 2020 Jul 14;10(44):26388–96. doi: 10.1039/d0ra04780e (PMC9122626; doi:10.1039/d0ra04780e)
Supplement: RA-010-D0RA04780E-s001 [file RA-010-D0RA04780E-s001.pdf]

Electronic Supplementary Material (ESI) for RSC Advances.
This journal is © The Royal Society of Chemistry 2020

'Targeting G-quadruplex DNA with synthetic dendritic peptide: modulation of the proliferation of human cancer cells'

Soumi Biswas,[a] Satyabrata Samui,[a] Apurba Kumar Das,[b] Sanjeev Pasadi,[c] K. Muniyappa,[c] Jishu Naskar[a]*

[a] Department of Biochemistry and Biophysics, University of Kalyani, Nadia, WB 741235, India

[b] Department of Chemistry, Indian Institute of Technology Indore, Khandwa Road, Indore 453552, India

[c] Department of Biochemistry, Indian Institute of Science, Bangalore, Karnataka 560 012, India

Contents

Chemical structure of the peptides

Synthetic procedure of the peptides

ESI-MS study

NMR study

Supplemental figures

References

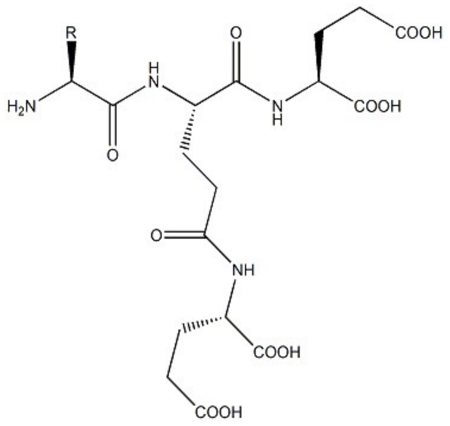

Figure S1: The chemical structure of the peptides. For peptide 1, R = $CH_2Ph(p{-}OH)$ and for peptide 2, R = $CH(CH_3)_2$

Experimental section:

The amino acid used in the preparation of peptides, coupling reagents dicyclohexylcarbodiimide (DCC), N-hydroxybenztriazole (HOBt), thiazole orange (TO) were purchased from SRL, India. The human telomeric quadruplex DNA (22AG) and the duplex DNA (ds26) were purchased from Eurofins, India.

Synthesis of peptides

Peptides are synthesized by conventional solution phase methods using racemization free fragment condensation strategy.[1] The Boc group has been used for N-terminal protection and the C-terminus -COOH group is protected as methylester. Coupling is mediated by dicyclohexylcarbodiimide and 1-hydroxybenzotriazole (DCC-HOBt). Deportation of OMe-group has performed using NaOH catalyzed saponification method and Boc-group by trifluoro aceticacid (TFA). All intermediates have been characterized by ^{1}H NMR spectroscopy and the final compounds are fully characterized by NMR spectroscopy, mass spectrometry.

Synthesis of Peptide 1

Synthesis of Boc-Tyr-OH: A mixture of 1, 4-dioxane, water and 1(N) NaOH were stirred in the round bottom flask (R.B). Then 3.64 gm tyrosine was added to this mixture. When tyrosine dissolved completely, the R.B was placed in ice cooled water bath and di-tertbutyldicarbonate (4.37 gm) was added. The solution was allowed to come at room temperature under stirring and it was continued for 6 hour. Then the solution was concentrated in *vacuum* to about 10 to15 mL, cooled in ice water bath, acidified with dilute solution of $KHSO_4$ to pH 2-3 (congo red) and covered with the layer of ethylacetate (about 10 mL). The aqueous phase was extracted with ethylacetate and this operation was done thrice. The ethylacetate extracts were pooled, washed with water and dried over anhydrous Na_2SO_4 and evaporated in *vacuum*. A white waxy material was obtained.

Yield: 4.35 gm, (77.10%)

Boc-Tyr-Glu(OMe)$_2$: 4.23 gm of Boc-Tyr-OH was dissolved in dry DMF in R.B and 2.02 gm of N-hydroxybenzotriazole (HOBt) was added to it. When HOBt was dissolved completely, R.B was placed in ice cooled water bath. 3-4 ml of ethylacetate containing L-Glu(OMe)$_2$, isolated from 4.83 gm of the corresponding methylester hydrochloride by neutralization with Na_2CO_3 and subsequent extraction with ethylacetate, was added to it and followed immediately by 3.09 gm of dicyclohexylcarbodimide (DCC). The reaction mixture was allowed to come to room temperature and stirring was continued for 48 h. The residue was taken up in ethylacetate (25 mL) and dicyclohexylurea (DCU) was filtered off. The organic layer was washed with 1(N) HCl, brine, 1(M) sodium carbonate and again with brine by the use of separating funnel. Then the ethylacetate extracts were pooled and dried over anhydrous sodium sulphate and evaporated in *vacuum.* A white material was obtained.

Yield: 4.85 gm, (73.8%).

^{1}H NMR (400 MHz, $CDCl_3$, δ): 6.87 (N<u>H</u>, 1H, *b*), 5.22 (N<u>H</u>, 1H, *b*), 7.00 (Tyr aromatic <u>H</u>, 2H, *d, J=8* Hz), 6.73 (Tyr aromatic <u>H</u>, 2H*, d, J= 8* Hz), 4.57-4.53 (C^{α}<u>H</u>, 1H, *m),* 4.32-4.30 (C^{α}<u>H</u>, 1H, *m*), 3.69 (-OC<u>H</u>$_3$, 3H, s), 3.65 (-OC<u>H</u>$_3$, 3H, s), 3.04-2.94 (Tyr C^{β}<u>H</u>, 2H, *m*), 2.35-1.90 (Glu C^{β}<u>H</u> and C^{γ}<u>H</u>, 4H, *m*), 1.41 (Boc C<u>H</u>$_3$, 9H, *s*).

Synthesis of Boc-Tyr-Glu(OH)$_2$: 4.5 gm Boc-Tyr-Glu(OMe)$_2$ was dissolved in MeOH at room temperature. At stirring condition, 2(N) NaOH was added gradually to it and the progress of saponification was monitored by thin layer chromatography (TLC). After 6h, methanol was removed under *vacuum*, the residue was taken in 10 mL of water, washed with diethyl ether, and pH of the aqueous layer was adjusted to 2-3 using $KHSO_4$ and it was extracted with ethylacetate. The extracts were pooled, dried over anhydrous sodium sulphate and evaporated in *vacuum*. A white solid material was obtained.

Yield: 3.75 gm (89 %).

^{1}H NMR (400 MHz, $CDCl_3$, δ): 7.83 (N<u>H</u>, 1H, *d, J*=8.0 Hz), 6.56 (N<u>H</u>, 1H, *d*, *J*=8.0 Hz), 6.80 (Tyr aromatic <u>H</u>, 2H, *d, J*=8.0 Hz), 6.40 (Tyr aromatic <u>H</u>, 2H*, d, J*=8.0 Hz), 4.03-3.98 (C^{α}<u>H</u>, 1H, *m)*, 3.87-3.82 (C^{α}<u>H</u>, 1H, *m*), 2.64-2.34 (Tyr C^{β}<u>H</u>, 2H, *m*), 1.78-1.53 (Glu C^{β}H and C^{γ}<u>H</u>, 4H, *m*), 1.06 (Boc C<u>H</u>$_3$, 9H, *s*).

Synthesis of ($C^{\delta 2}$)-[Boc-Tyr-Glu-Glu(OMe)$_2$]-Glu(OMe)$_2$: 3.30 gm Boc-Tyr-Glu(OH)$_2$ was dissolved in dry DMF in R.B. 1.08 gm of HOBt was added to it. When HOBt dissolved completely, R.B was placed in ice cooled water bath. 3-4 ml of ethylacetate containing L-Glu(OMe)$_2$, isolated from the corresponding methylester hydrochloride by neutralization with Na_2CO_3 and subsequent extraction with ethylacetate, was added to it and followed immediately by 1.65 gm of dicyclohexylcarbodimide (DCC). The reaction mixture was allowed to come to room temperature and stirring was continued for 48 h. The residue was taken up in ethylacetate and dicyclohexylurea (DCU) was filtered off. The organic layer was washed with 1(N) HCl, brine, 1(M) sodium carbonate and again brine by the use of separating funnel. Then ethylacetate extracts were pooled and dried over anhydrous sodium sulphate and evaporated in *vacuum.* Finally a white material was obtained.

Purification of $C^{\delta 2}$-[Boc-Tyr-Glu-Glu(OMe)$_2$]-Glu(OMe)$_2$: Purification of $C^{\delta 2}$-[Boc-Tyr-Glu-Glu(OMe)$_2$]-Glu(OMe)$_2$ was done by column chromatography using chloroform : methanol (95:5) as a mobile phase. Silica gel 100-200 mesh was used as the stationary phase.

Yield: 3.18 gm (54.6%).

^{1}H NMR (400 MHz, $CDCl_3$, δ): 6.96 (N<u>H</u>, 1H, *d, J*=8 Hz), 6.91 (Tyr aromatic <u>H</u>, 2H, *d, J*=8 Hz), 6.69 (N<u>H</u>, 1H, *d, J*=8 Hz), 6.66 (Tyr aromatic <u>H</u>, 2H*, d, J*= 8 Hz), 5.17 (N<u>H</u>, 1H, *d, J*=8 Hz), 5.09 (N<u>H</u>, 1H, *d, J*=8 Hz), 4.45-4.21 (C^{α}<u>H</u>, 4H, *m)*, 3.67(-OC<u>H</u>$_3$, 3H, *s*), 3.65 (-OC<u>H</u>$_3$, 3H, *s*), 3.61 (-OC<u>H</u>$_3$, 3H, *s*), 3.59 (-OC<u>H</u>$_3$, 3H, *s*), 2.93-2.83 (Tyr C^{β}<u>H</u>, 2H, *m*), 2.40-1.81 (Glu C^{β}H and C^{γ}<u>H</u>, 12 H, *m*), 1.34 (Boc C<u>H</u>$_3$, 9H, *s)*.

$C^{\delta 2}$-[H-Tyr-Glu-Glu(OH)$_2$]-Glu(OH)$_2$: 2.9 gm of ($C^{\delta 2}$)-[Boc-Tyr-Glu-Glu(OMe)$_2$]-Glu(OMe)$_2$ was saponified and then trifluoroacetic acid (TFA) was added, removal of Boc group was monitored by TLC. After 4h, TFA was removed under *vacuum*. The residue was taken in water (20 ml). The aqueous portion was evaporated in *vacuum* to yield peptide **1** as white solid.

Yield = 1.25 g (68.5%).

ESI-MS data (calculated): $(M)^+$ = 568.5305 and $(M+H)^+$ = 569.2050.

Observed: $(M)^+$ = 568.9980 and $(M+H)^+$ = 570.0118

^{1}H NMR (500 MHz, DMSO-*d$_6$*, δ): 8.16 (N<u>H</u>, 1H, *d, J*=10 Hz), 7.89 (N<u>H</u>, 1H, *d, J*=15 Hz), 7.08 (Tyr aromatic <u>H</u>, 2H, *d, J*=10 Hz), 7.00 (N<u>H</u>, 1H, *d, J*=15 Hz), 6.74 (Tyr aromatic <u>H</u>, 2H*, d, J*= 10 Hz), 4.36-4.29 (C^{α}<u>H</u>, 1H, *m)*, 4.09-4.02 (C^{α}<u>H</u>, 2H, *m)*, 3.66-3.54 (C^{α}<u>H</u>, 1H, *m)*, 3.00-2.64 (Tyr C^{β}<u>H</u>, 2H, *m*), 2.28-1.77 (Glu C^{β}H and C^{γ}<u>H</u>, 12 H, *m*).

^{13}C NMR (125 MHz, DMSO-*d$_6$*, δ): 159.77, 159.72, 159.45, 159.37, 159.13, 159, 158.82, 121.71, 118.85, 115.69, 112.83, 59.87, 57.62, 52.24, 51.57, 31.67, 30.51, 29.48, 28.44, 18.80, 17.93

Synthesis of Peptide 2

Synthesis of Boc-Val-OH: A mixture of 1, 4-dioxane, water and 1(N) NaOH were stirred in the round bottom flask. Then 2.34 gm valine was added to this mixture. When valine dissolved completely, the R.B was placed in ice cooled water bath and di-tertbutyldicarbonate (4.37 gm) was added. The solution was allowed to come at room temperature under stirring and it was continued for 12 hour. Then the solution was concentrated in *vacuum* to about 10 to15 mL, cooled in ice water bath, acidified with dilute solution of $KHSO_4$ to pH 2-3 (congo red) and covered with the layer of ethylacetate (about 10 mL). The aqueous phase was extracted with ethylacetate and this operation was done thrice. The ethylacetate extracts were pooled, washed with water and dried over anhydrous Na_2SO_4 and evaporated in *vacuum*. A white waxy material was obtained.

Yield: 3.15 gm, (72.55%).

Boc-Val-Glu(OMe)$_2$: 3.04 gm of Boc-Val-OH was dissolved in dry DMF in R.B and 1.89 gm of N-hydroxybenzotriazole (HOBt) was added to it. When HOBt was dissolved completely, R.B was placed in ice cooled water bath. 3-4 ml of ethylacetate containing L-Glu(OMe)$_2$, isolated from the corresponding methylester hydrochloride by neutralization with Na_2CO_3 and subsequent extraction with ethylacetate, was added to it and followed immediately by 2.88 gm of dicyclohexylcarbodimide (DCC). The reaction mixture was allowed to come to room temperature and stirring is continued for 48 h. The residue was taken up in ethylacetate (25 mL) and dicyclohexylurea (DCU) was filtered off. The organic layer was washed with 1(N) HCl, brine, 1(M) sodium carbonate and again with brine by the use of separating funnel. Then the ethylacetate extracts were pooled and dried over anhydrous sodiumsulphate and evaporated in *vacuum.* A white material was obtained.

Yield: 3.85 gm, (73.5%).

^{1}H NMR (400 MHz, $CDCl_3$, δ): 6.860 (N<u>H</u>, 1H, *d, J*=6 Hz), 5.09 (N<u>H</u>, 1H, *d, J*=8.4 Hz), 4.64-4.59 (C^{α}<u>H</u>, 1H, *m)*, 4.24-4.20 (C^{α}<u>H</u>, 1H, *m*), 3.74 (-OC<u>H</u>$_3$, 3H, *s*), 3.67 (-OC<u>H</u>$_3$, 3H, *s*), 2.47-2.35 (Glu C^{γ}H, 2H, m) 2.27-2.10 (C^{β}<u>H</u>, 2H, *m*), 2.06-1.98 (Val C^{β}<u>H</u>, 1H, *m*), 1.44 (Boc C<u>H</u>$_3$, 9H, *s)*, 0.98-0.89 (Val C^{γ}<u>H</u>, 6H, *m*).

Synthesis of Boc-Val-Glu(OH)$_2$: 3.56 gm Boc-Val-Glu(OMe)$_2$ was dissolved in MeOH at room temperature. At stirring condition, 2 (N) NaOH was added gradually to it and the progress of saponification was monitored by thin layer chromatography (TLC). After 6h, methanol was removed under *vacuum*, the residue was taken in 10 mL of water, washed with diethyl ether and pH of the aqueous layer was adjusted to 2-3 using $KHSO_4$ and it was extracted with ethylacetate. The extracts were pooled, dried over anhydrous sodium sulphate and evaporated in *vacuum*. A white solid material was obtained.

Yield: 3.10 gm (94.21 %).

^{1}H NMR (400 MHz, $CDCl_3$, δ) **:** 5.73 (Glu N<u>H</u>, 1H, *d*, *J*=8.8 Hz), 5.18 (Val N<u>H</u>, 1H, *d, J*=8.8 Hz), 4.59-4.56 (C^{α}<u>H</u>, 1H, *m)*, 4.25-4.22 (C^{α}<u>H</u>, 1H, *m*), 2.43-2.39 (Glu C^{γ}<u>H</u>, 2H, *m*), 2.22-2.17 (Glu C^{β}<u>H</u>, 2H, *m*), 1.98-1.94 (Val C^{β}<u>H</u>, 1H, *m*), 1.40 (Boc C<u>H</u>$_3$, 9H, *s)*, 0.95-0.89 (Val C^{γ}<u>H</u>, 6H, *m*).

Synthesis of $C^{\delta 2}$-[Boc-Val-Glu-Glu(OMe)$_2$]-Glu(OMe)$_2$: 2.90 gm of Boc-Val-Glu(OH)$_2$ was dissolved in 5 mL of dry DMF in R.B and 1.13 gm of HOBt was added to it. When HOBt dissolved completely, R.B was placed in ice cooled water bath. 3-4 ml of ethylacetate containing L-Glu(OMe)$_2$, isolated from the corresponding methyl ester hydrochloride by neutralization with Na_2CO_3 and subsequent extraction with ethylacetate, was added to it and followed immediately by 1.73 gm of dicyclohexylcarbodimide (DCC). The reaction mixture was allowed to come to room temperature and stirring was continued for 48 h. The residue was taken up in ethylacetate (25 mL) and dicyclohexylurea (DCU) was filtered off. The organic layer was washed with 1(N) HCl, brine, 1(M) sodium carbonate and again with brine by the use of separating funnel. Then the ethylacetate extracts were pooled and dried over anhydrous sodium sulphate and evaporated in *vacuum.* A white material was obtained.

Purification of ($C^{\delta 2}$)-[Boc-Val-Glu-Glu(OMe)$_2$]-Glu(OMe)$_2$: Purification of **($C^{\delta 2}$)-[Boc-Val-Glu-Glu(OMe)$_2$]-Glu(OMe)$_2$** was done by column chromatography using chloroform:methanol (95:5) as a mobile phase. Silica gel 100-200 mesh was used as a stationary phase.

Yield: 3.17 gm (57.27%).

^{1}H NMR (400 MHz, $CDCl_3$, δ) **:** 7.93 (N<u>H</u>, 1H, *d, J*=8 Hz), 7.65 (N<u>H</u>, 1H, *d, J*=8.4 Hz), 6.76 (N<u>H</u>, 1H, *d, J*=6.4 Hz), 5.14 (N<u>H</u>, 1H, *d, J*=8 Hz), 4.73-4.64 (C^α<u>H</u>, 2H, *m)*, 4.32-4.26 (C^α<u>H</u>, 1H, *m)*, 3.96-3.94 (C^α<u>H</u>, 1H, *m)*, 3.76(-OC<u>H</u>$_3$, 3H, s), 3.73 (-OC<u>H</u>$_3$, 3H, s), 3.68 (-OC<u>H</u>$_3$, 3H, s), 3.66 (-OC<u>H</u>$_3$, 3H, s), 2.49-2.00 (Glu C^βH and C^γ<u>H</u>, 12H, *m*), 1.98-1.95 (Val C^β<u>H</u>, 1H, *m*), 1.43 (Boc C<u>H</u>$_3$, 9H, *s)*, 0.98-0.88 (Val C^γ<u>H</u>, 6H, *m*).

$C^{\delta 2}$-[H-Val-Glu-Glu(OH)$_2$]-Glu(OH)$_2$: 2.70 gm of **($C^{\delta 2}$)-[Boc-Val-Glu-Glu(OMe)$_2$]-Glu(OMe)$_2$** was saponified and then trifluoroacetic acid (TFA) was added, removal of Boc group was monitored by TLC. After 4 h, TFA was removed under *vacuum*. The residue was taken in water (20 ml). The aqueous portion was evaporated in *vacuum* to yield peptide **2** as white solid.

Yield = 1.35 g (65.28 %)

^{1}H NMR (500 MHz, DMSO-d_6, δ): 8.60 (N<u>H</u>, 1H, *d, J*=10 Hz), 8.41 (N<u>H</u>, 1H, *d, J*=10 Hz), 8.15 (N<u>H</u>, 1H, *d, J*=5 Hz), 4.46-4.25 (C^α<u>H</u>, 3H, *m)*, 3.70-3.67 (C^α<u>H</u>, 1H, *m)*, 2.43-1.78 (Glu C^βH and C^γ<u>H</u>, 12 H, m), 1.03-1.00 (Val C^β<u>H</u>, 1H, *m*), 0.99-0.97 (Val C^γ<u>H</u>, 6H, *d*).

^{13}C NMR (125 MHz, DMSO-d_6, δ): 174.38, 174.15, 173.42, 171.24, 168.18, 158.86, 158.44, 57.74, 57.67, 52.29, 51.62, 30.39, 30.31, 27.97, 26.82, 26.65, 26.62, 18.71, 18.16

ESI-MS data (calculated): $(M)^+$ = 504.2068 and $(M+H)^+$ = 505.2068.

Observed: $(M+H)^+$ = 505.2451

NMR experiments

NMR spectra of final compounds are recorded on 500 MHz spectrometer in DMSO-d_6. Other intermediates NMR studies are carried out on a Brüker DPX 400 MHz spectrometer in $CDCl_3$.

Synthesis of G-quadruplex and double stranded DNA

To prepare the stock solution of ds26 and 22AG tris-HCl buffer containing 100 mM of NaCl have been used and the pH was adjusted at 7.3. The quadruplex and duplex structure were prepared by heating the self complementary strand at 90°C for 5 min in Na^+ buffer followed by a slow cooling over 6 h then the solutions were stored at 277.15 K for 48 hours.[2] Concentrated working stock solution of the synthesized peptides was also prepared by dissolving the peptides in the same buffer and the pH was adjusted at 7. All reagents used here were analytical grade. The concentrations of the oligonucleotides were determined from the information provided by the supplier.

Gel mobility shift assay: Gel mobility shift assay was performed incubating 30 μM of quadruplex DNA with increasing concentration of both peptide 1 and peptide 2 for 30 minutes at room temperature. The products were then resolved on 15% of native polyacrylamide gel [29:1 acrylamide/bis(acrylamide)] prepared by polymerizing acrylamide in 5X TBE buffer. The gel was visualized by staining with ethidium bromide (EtBr) and analyzed by using gel doc (ChemiDoc XRS+, Biorad).

Isothermal titration calorimetry: Isothermal titration calorimetry (ITC) experiments were carried out on a TA nano ITC instrument. Before starting the experiment, peptide and DNA solutions were degassed for 10 minutes in the degassing chamber.

Fluorescence titration experiment: Fluorescence spectra of peptide 1 (15 μM) were recorded on a PPI-QM40 Spectrofluorimeter at room temperature. Excitation was fixed at 275 nm and emission spectra were recorded in the range from 280 to 400 nm. G4 DNA concentrations were varied from 0 to 18.16 μM.

Thiazole orange displacement assay: Thiazole orange (TO) displacement experiments were performed with prefolded G4 DNA (0.25 μM) and TO (0.50 μM) complex. Addition of increasing amount of peptide 1 and peptide 2 (from 0 to 14.5 μM) were followed by a 2 minute equilibration period before the fluorescence spectra were recorded upon excitation at 501 nm at 298K. The fluorescence area (FA, 510-750 nm), converted in percentage displacement (PD, with PD = 100—([F_A/F_{Ao}] × 100), F_{Ao} and F_A indicates before and after addition of the peptide 1 and peptide 2 respectively. Then the PD values were plotted against the concentration of added peptide 1 and peptide 2. The TO displacement data were then quantified by DC50 values, which represent the concentration of the peptides required to displace 50% of TO from the G4 DNA.

Time correlated single photon counting (TCSPC): Fluorescence lifetimes were measured by the Time Correlated Single Photon Counting (TCSPC) technique on a FluoroCube- 01-NL spectrometer (Horiba Jobin Yvon). Laser-diode was used as an excitation source and peptide 1 was excited at 275 nm.

Circular dichroism spectroscopy study: Circular dichroism (CD) spectra were recorded on a JASCO J-815 spectropolarimeter using a cuvette of 1 mm path length at room temperature. Each spectrum was the average of three scans, and the scan range was taken from 220-320 nm. The CD study was performed keeping the concentration of G4 DNA constant while varying the concentration of peptide 1 and peptide 2.

CD melting study: The melting point study of free G4 DNA and the complex formation of G4 DNA with both peptide 1 and peptide 2 were done using the peltier attached JASCO J-815 spectropolarimeter from the temperature range (30-80)˚C.

Cell Viability Assay: The cells were seeded in 12-well plates and grown before treatment to obtain >70% confluency and exposed to either various concentrations of peptide 1. After 72 h of incubation at 37 °C in a humidified atmosphere of 5% CO2, the medium was replaced with the fresh medium containing 10% FBS. Then, 0.5 mg/mL methyl thiazolyl tetrazolium (MTT) reagent was added. The formazan crystals were dissolved in DMSO,

and the absorbance values were recorded using the ELISA plate reader. The percentage of cell viability was calculated from the absorbance values. All ligand doses were tested in triplicate.

Long-Term Cell Proliferation assay: HeLa and HEK 293 cells were grown in six-well tissue culture plates at a seeding density of 5.0×10^4 per cells per well. The cells were treated with the subcytotoxic concentration of 850 μM and incubated with the cells for 5 days. At the fifth day, the cells were counted using Trypan blue, reseeded in new wells with half the population of cells following which, treatment was given for another 5 days. In this way, the proliferation of the cells was monitored over a period of 15 days. Plots of cell population versus time (days) depict the anti-proliferative activity.

Cell Cycle Analysis: The cell cycle analysis was performed on HeLa and HEK 293 stained with propidium iodide (PI). The peptide treated cells were fixed with 70% ethanol, treated with RNase, and stained with PI (1μg/mL) for a period of 30 min followed by analysis on a FACS machine. The population of cells at the sub-G1 region (% apoptotic cells) was determined by CytExpert 2.0 software.

TRAP assay: TRAP assay was done using the protocol as described in the literature *Chem. Asian J.*, **2016**, *11*, 2542– 2554.[2] Briefly, the TRAP assay was performed using the three steps protocol 1. Primer elongation by telomerase and addition of ligand. 2. Subsequent removal of the ligand. 3. PCR amplification of the products of telomerase elongation.

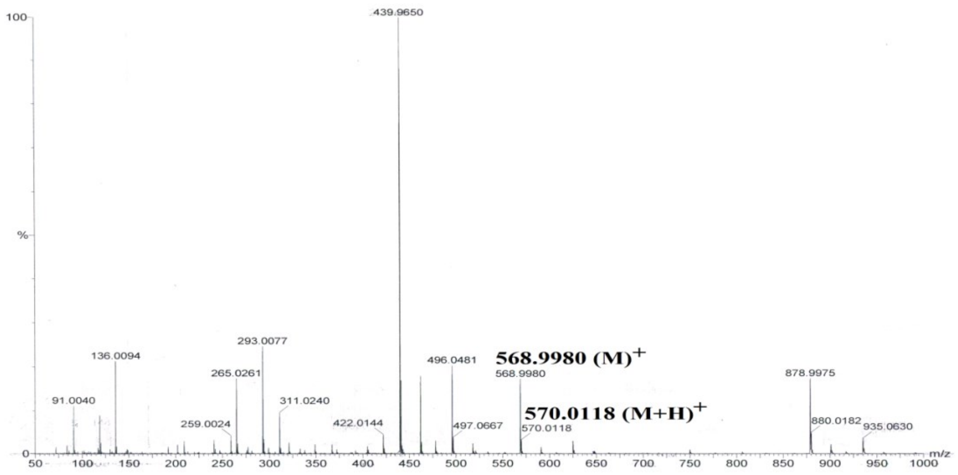

Figure S2: HRMS spectrum of peptide **1.**

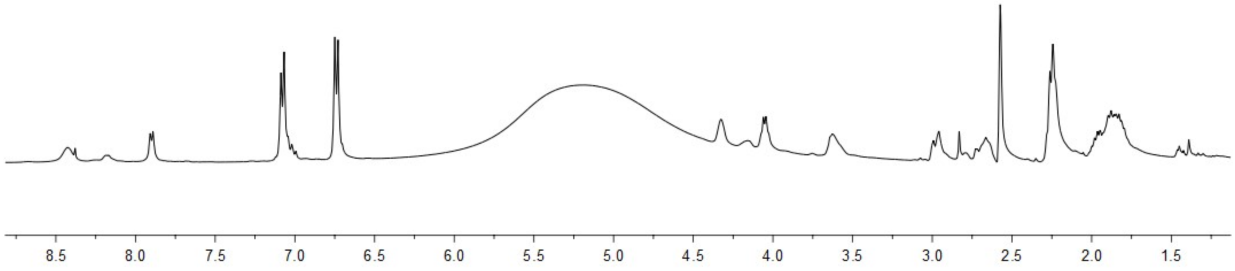

Figure S3: ^{1}H NMR spectrum of peptide 1(500 MHz, DMSO-d_6 , 300 K).

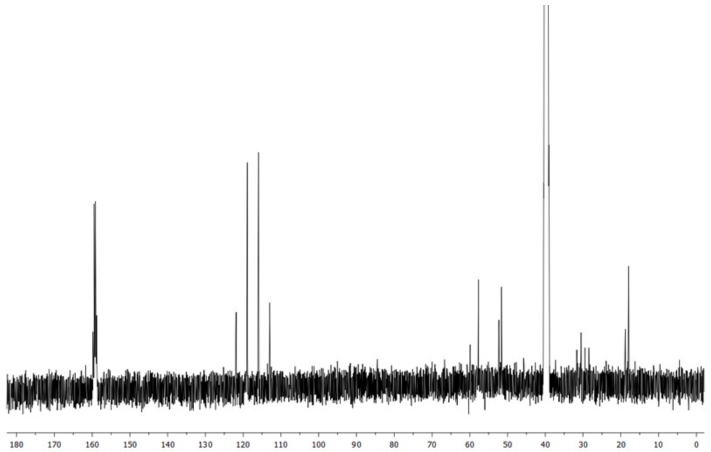

Figure S4: ^{13}C NMR spectrum of peptide 1.

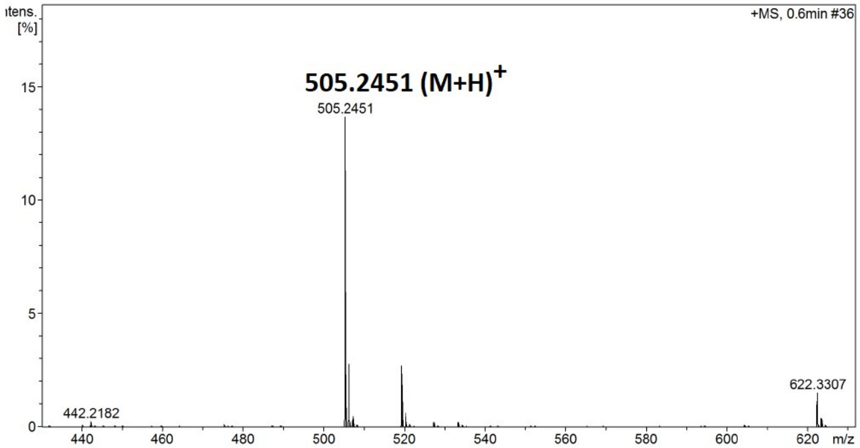

Figure S5: HRMS spectrum of peptide 2.

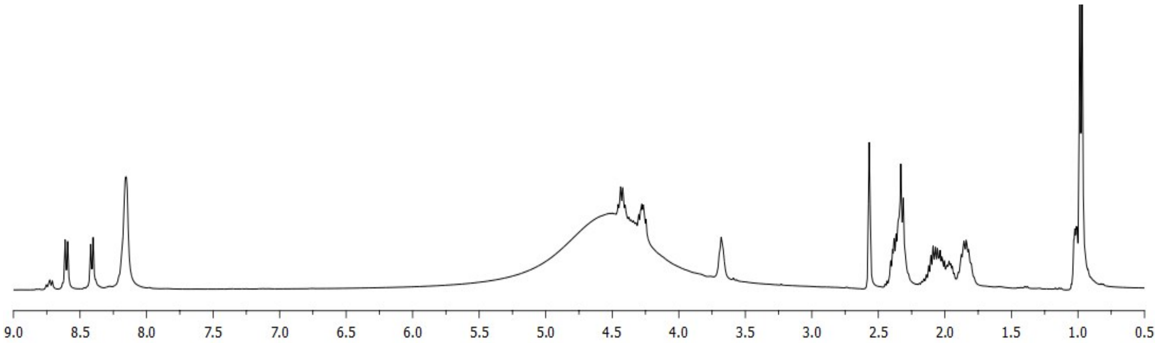

Figure S6: ^{1}H NMR spectrum of peptide 2 (500 MHz, DMSO-d_6 , 300 K).

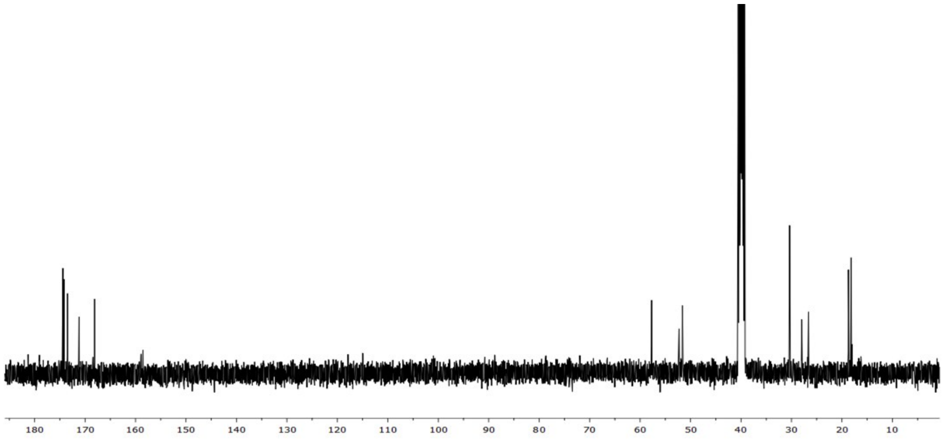

Figure S7: ^{13}C NMR spectrum of peptide 2.

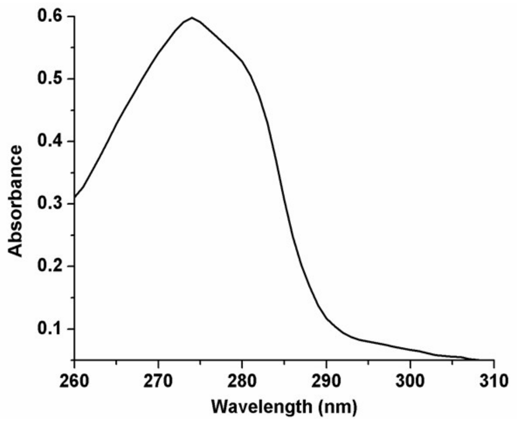

Figure S8: The UV spectra of peptide 1 at 30 μM concentration.

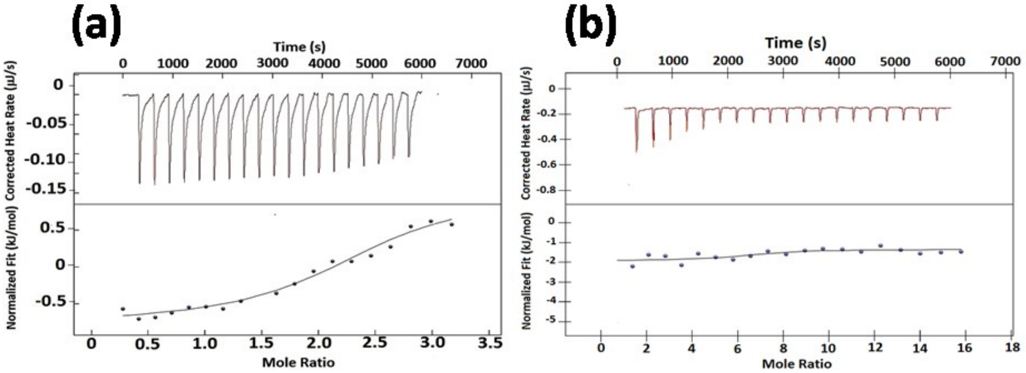

Figure S9: ITC profiles of titration of (a) peptide 1 with ds-DNA, (b) peptide 2 with ds-DNA at T = 298.15 K. The top panels of both (a) and (b) represent the amount of heat generated per sequential injection of peptides into ds-DNA solution, and the bottom panels show the integrated heat data after correction of heat dilution against the molar ratio of peptide to ds-DNA. The solid lines represent the best fitted plot.

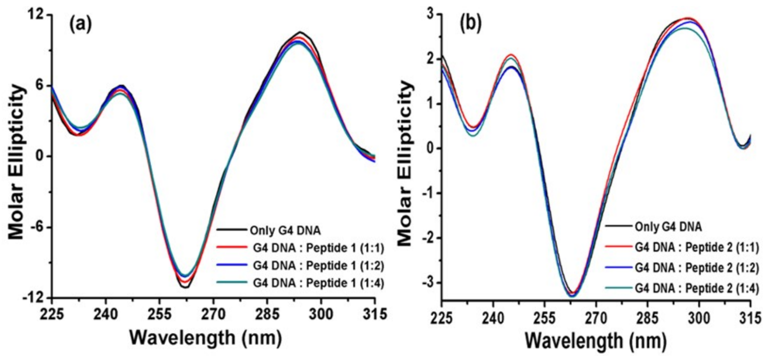

Figure S10: CD spectra of human telomeric G4 DNA in the presence of various concentrations of (a) peptide 1 and (b) peptide 2 .

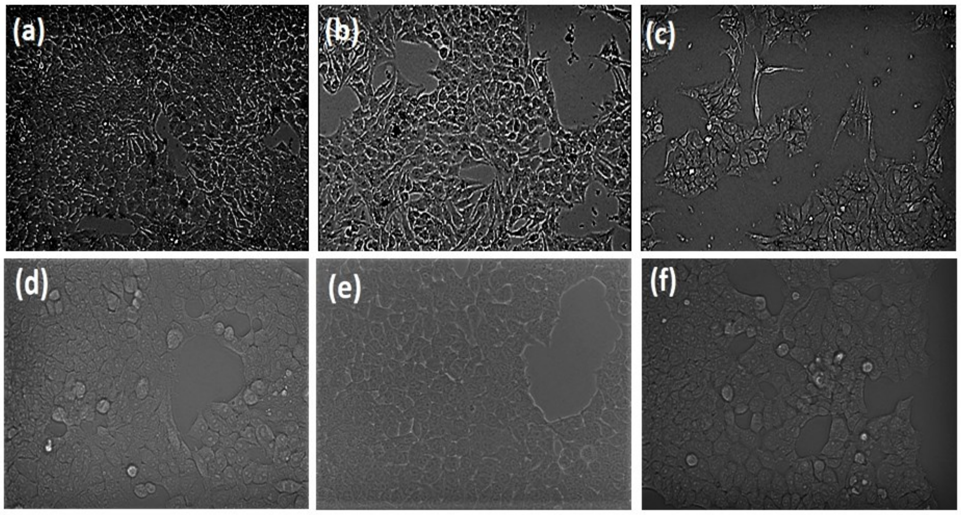

Figure S11: Phase contrast microscopic images showing cell morphology after treatment with peptide 1. (a), (d) untreated control. (b), (e) treatment with 1 mM of peptide 1. (c), (f) treatment with 1.5 mM of peptide 1. Upper and lower panels represent morphology of HeLa and HEK 293 cells respectively.

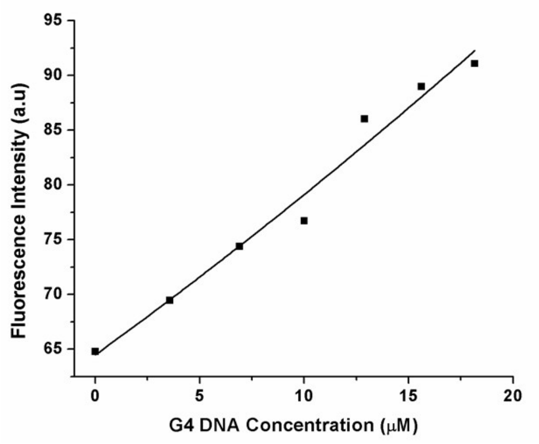

Figure S12: Fluorescence titration plot. The plot shows the changes in fluorescence intensity of peptide upon gradual addition of G4 DNA.

References:

[1]. Bodanszky M, Bodanszky A, The practice of peptide synthesis. *Springer*: *New York* (1984), 1–282.

[2]. A. Ali, M. Kamra, S. Roy, K. Muniyappa, S. Bhattacharya, *Chem. Asian J.*, **2016**, *11*, 2542– 2554.
